# Supplementary material for: Oestrogen Non-Genomic Signalling is Activated in Tamoxifen-Resistant Breast Cancer
Source: Int J Mol Sci. 2019 Jun 5;20(11):2773. doi: 10.3390/ijms20112773 (PMC6600329; doi:10.3390/ijms20112773)
Supplement: Supplementary file 1 [file ijms-20-02773-s001.pdf]

Supplementary information

Oestrogen non-genomic signalling is activated in tamoxifen-resistant breast cancers

Coralie Poulard, Julien Jacquemetton, Olivier Trédan, Pascale A. Cohen, Julie Vendrell, Sandra E. Ghayad, Isabelle Treilleux, Elisabetta Marangoni and Muriel Le Romancer

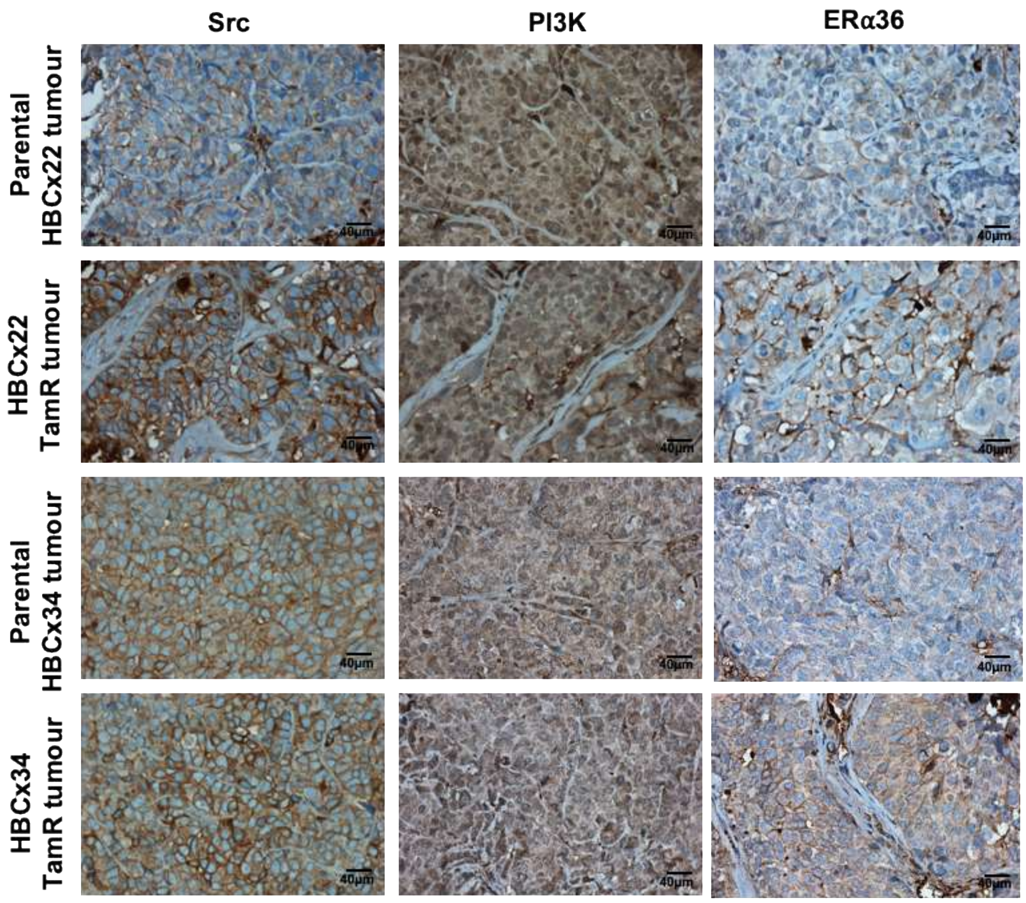

Supplementary Fig 1. Study of Src, PI3K and ERα-36 expression in parental and resistant PDXs. IHC experiments were performed on tumours embedded in paraffin using the corresponding antibodies.

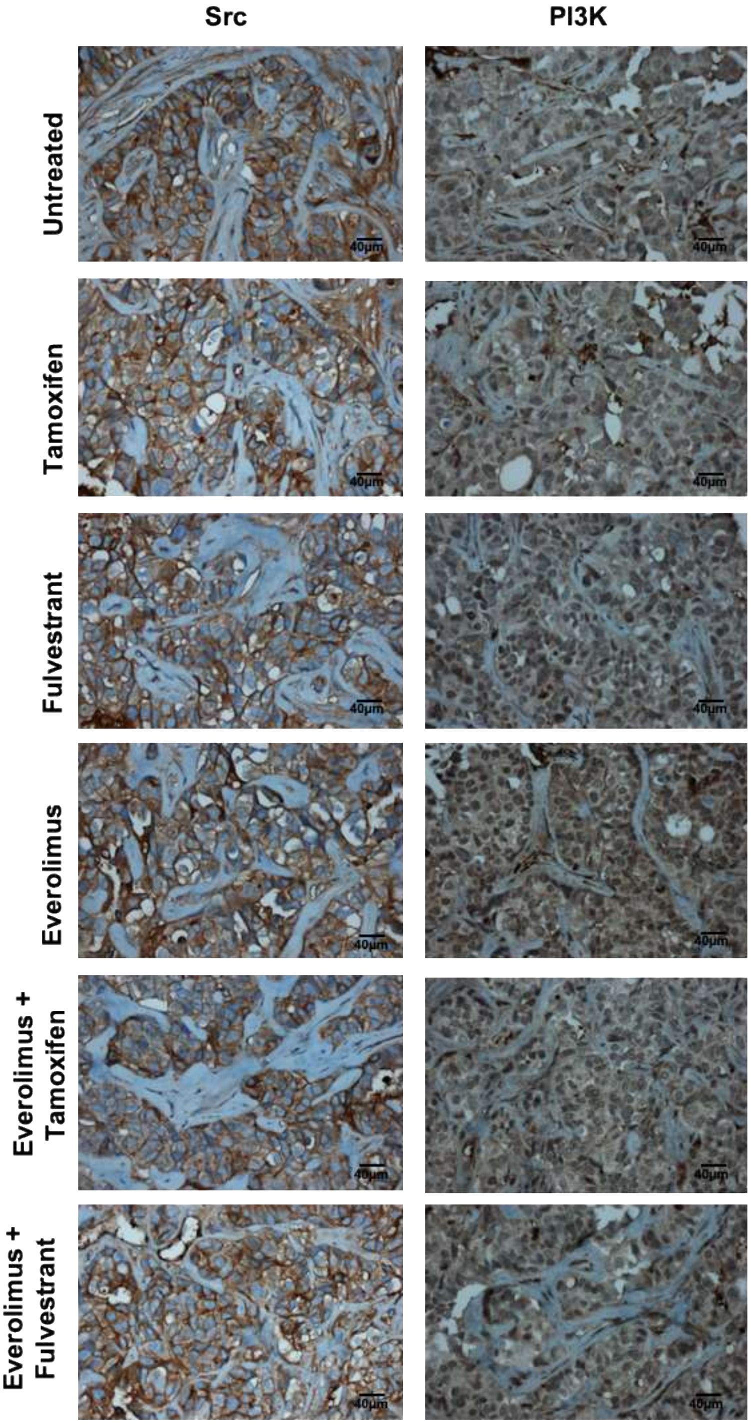

Supplementary Fig 2. IHC analysis of treated tumours. Representative sections of TMA obtained from therapeutic experiments on HBCx22 TamR.

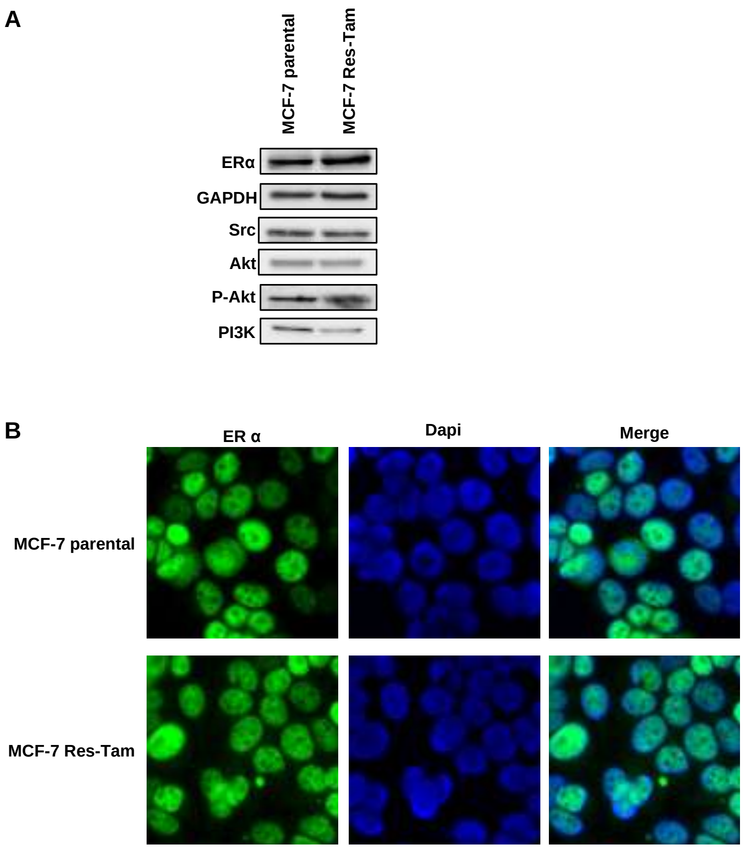

Supplementary Fig 3. Study of the expression of the members of the oestrogen non genomic pathway in parental and tamoxifen resistant MCF-7 cells. (**A**) Cell lysates of parental and tamoxifen resistant MCF-7 cells were analysed for ERα, Src, PI3K, P-Akt, Akt and GAPDH expression by Western blot. (**B**) Methanol-fixed cells were stained with an anti-ERα antibody. The nuclei were counterstained with mounting medium containing DAPI (blue) (x60 magnification).
